# Complexity and Diversity of the NKR-P1:Clr (*Klrb1*:*Clec2*) Recognition Systems

**DOI:** 10.3389/fimmu.2014.00214

**Published:** 2014-06-02

**Authors:** Christina L. Kirkham, James R. Carlyle

**Affiliations:** ^1^Department of Immunology, University of Toronto, Sunnybrook Research Institute, Toronto, ON, Canada

**Keywords:** natural killer cell, innate immunity, C-type lectin-like, Nkrp1, CD161, Clr, Ocil, LLT1

## Abstract

The NKR-P1 receptors were identified as prototypical natural killer (NK) cell surface antigens and later shown to be conserved from rodents to humans on NK cells and subsets of T cells. C-type lectin-like in nature, they were originally shown to be capable of activating NK cell function and to recognize ligands on tumor cells. However, certain family members have subsequently been shown to be capable of inhibiting NK cell activity, and to recognize proteins encoded by a family of genetically linked C-type lectin-related ligands. Some of these ligands are expressed by normal, healthy cells, and modulated during transformation, infection, and cellular stress, while other ligands are upregulated during the immune response and during pathological circumstances. Here, we discuss historical and recent developments in NKR-P1 biology that demonstrate this NK receptor–ligand system to be far more complex and diverse than originally anticipated.

## Introduction

Natural killer (NK) cells are innate lymphocytes that recognize and respond to a variety of different pathological target cells via cytotoxicity and secretion of type I helper cytokines, most notably IFN-γ. However, they can also secrete TNF-α, GM-CSF, and chemokines, to initiate crosstalk with the adaptive immune system. Target cell recognition is mediated by a variety of receptors on NK cells that detect specific cognate ligands, which in turn are differentially expressed on pathological versus normal cells (1). Healthy cells broadly express a number of inhibitory ligands (including MHC-I molecules), which are frequently lost during pathological transformation, infection, or cell stress; this has been termed “missing-self” recognition (1, 2). On the other hand, most stimulatory ligands are minimally expressed by healthy cells, but are strongly upregulated during cellular pathologies; this phenomenon has been termed “induced-self” recognition (3). Typically, both missing-self and induced-self recognition events operate simultaneously to shift the balance from NK cell tolerance to induction of NK cell activation, via an integration of target cell recognition signals (3, 4).

**Figure 1:**
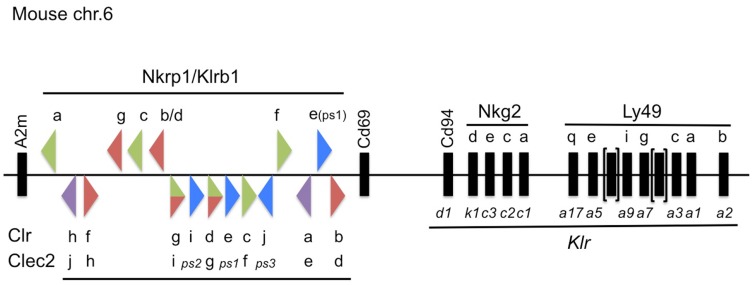
**Updated map of the NKC on mouse chromosome 6, highlighting the Nkrp1/*Klrb1* and Clr/*Clec2* receptor–ligand system**. The mouse Nkrp1 receptor genes (official gene nomenclature, *Klrb1*) are highlighted at the top, with the Clr ligand genes (official gene nomenclature, *Clec2*) denoted below, located between the *A2m* and *Cd69* genes (17). The triangles depict the gene orientation (plus or minus strand) and their color signifies the known or suspected function of the gene products in regulating NK cell activity, as follows: co-stimulatory (green); co-inhibitory (red); bifunctional (green/red); pseudogene (*ps*, blue); or unknown (purple). Other select NK receptor genes located telomeric to *Cd69* are also depicted with common and official (*Klr*) gene nomenclatures. Not to scale.

Many NK cell receptors are encoded by genes linked to the NK gene complex (NKC), located on chromosome 6 in mice, chromosome 4 in rats, and chromosome 12 in humans (5–7) (Figure 1). The NKC is also conserved among several other species, including dogs (7), cattle (where it is split between two chromosomes) (7), and chickens (where it is genetically linked to the MHC region) (8). However, a number of NK cell receptor gene families are linked to the leukocyte receptor cluster (LRC), located on mouse chromosome 7, rat chromosome 1, and human chromosome 19 (9–12). There are also numerous other NK cell receptors encoded in various regions in the genome, including the SAP/SLAM family of receptors found on mouse and human chromosome 1 (13, 14), the natural cytotoxicity receptors (*NCR1, 2, 3*) (15, 16), and others. Within these regions, most NK cell receptors can be broadly classified into two structurally divergent categories: immunoglobulin-like receptors (e.g., KIR, NCR) and C-type lectin-like receptors (e.g., KLR, Ly49).

Among the earliest identified group of NK cell receptors is the NKR-P1 family (encoded by the *Klrb1* genes; centromeric to *Cd69* in the NKC) [Reviewed in Ref. (17)] (Figure 1). This family of receptors is somewhat unique within the NKC, because their ligands are other C-type lectin-related proteins (Clr; encoded by the *Clec2* genes), and the *Clr/Clec2* loci are genetically interspersed amongst the *Nkrp1/Klrb1* receptor genes themselves (18–20) (Figure 1). Although a number of functional interactions have been demonstrated to date between different NKR-P1 receptors and Clr ligands in mice (21), rats (22), and humans (23), many of the receptor–ligand interactions remain unknown, and most have unknown function. This review will outline the historical and recent discoveries surrounding the NKR-P1:Clr systems in rodents and humans, and provide an update on their nomenclature, as well as known expression, structure, and function.

## Historical Perspective

### Discovery of the NKR-P1 (Klrb1/CD161/Ly-55/Ly-59/Clec5b) receptors

The first receptor identified to be selectively expressed by NK cells was the mouse NK1 alloantigen, which was discovered by Glimcher et al. in 1977 and found to be differentially expressed across mouse strains (24) [reviewed in Ref. (17)]. The development of a specific monoclonal antibody (PK136 mAb) facilitated its designation as the NK1.1 antigen, permitting the detection and purification of NK cells in select inbred mouse strains (25, 26). Subsequently, the NK1.1 antigen was shown to possess activating function (27, 28), providing direct evidence that NK cells express receptors that may be capable of recognizing cognate ligands on target cells (29). However, the identity of the NK1.1 antigen would remain unknown for several years (30).

In 1989, Chambers et al. generated a mAb (3.2.3) against a cell surface antigen present at high density on rat NK cells and purified rat lymphokine-activated killer (LAK) cells (31). The 3.2.3 antibody was shown to induce redirected NK cell cytotoxicity against FcR^+^ targets, as well as exocytosis of NK cell cytolytic granules, classifying it as an activating receptor. They called the antigen NKR-P1A (32). Since ligation of mouse NK1.1 and rat NKR-P1A both induced NK cell-mediated cytotoxicity, the hypothesis arose that they could represent homologous structures (28).

Consequently, Giorda et al. screened a B6-strain mouse LAK cDNA library using the rat NKR-P1A cDNA, and identified three mouse NKR-P1 homologs, which they called NKR-P1A (clone 2), NKR-P1B (clone 34), and NKR-P1C (clone 40) (33). The cloned sequences corresponding to the mouse NKR-P1 cDNA were found to exist in different sizes, suggestive of alternative splicing. Overall, these cDNA shared between 61–87% identity at the amino acid level with rat NKR-P1A, with high similarity existing in the extracellular lectin-like region, including several C residues and *N*-linked glycosylation sites. The discovery and designation of the NKC in mice in 1991 showed that the NKR-P1 receptor loci were distinct from the Ly49 receptor loci, despite their common expression and structural similarity (34). Importantly, however, their genetic linkage demonstrated that a specific location on mouse chromosome 6 was dedicated to NK cell function (5). With the physical mapping of the NKR-P1 genes and the locus encoding the NK1.1 antigen to the same region of mouse chromosome 6, along with their similar expression, structure, and function, it became increasingly likely that the NK1.1 antigen belonged to the NKR-P1 family, and this was formally demonstrated by Ryan et al. in 1992, via expression cloning of the mouse *Nkrp1c^B6^* cDNA using PK136 mAb (35).

However, it later became clear that the strain-dependent expression of the NK1.1 antigen was not only due to allelic expression of the *Nkrp1c^B6^* gene product. In 1999, two groups demonstrated that *Nkrp1b* gene products from the Swiss-NIH and SJL strains also reacted with the NK1.1 mAb, PK136 (36, 37). Furthermore, the NKR-P1B^Sw/SJL^ receptors inhibited NK cell function rather than activating NK cells like NKR-P1C. In these studies, the cloned NKR-P1B^B6^ (NKR-P1D) gene products did not react with NK1.1 mAb, nor did the NKR-P1C^Sw/SJL^ gene products, making it unclear whether they were alleles of existing genetic loci or new genes. In any case, these results demonstrated that polymorphisms exist at both the mouse *Nkrp1b* and *Nkrp1c* loci (see below).

In 2001, a BAC contig of the *Nkrp1* gene cluster in the B6 mouse strain allowed for the identification of several new genomic sequences, including *Nkrp1d* (*Nkrp1b*^B6^), *Nkrp1e*, and *Nkrp1f* (38). The *Nkrp1d* sequence is 90% similar to that of *Nkrp1b*, and likely represents an allele of the *Nkrp1b* locus, rather than a new gene, since the coding sequence resembles that of the cloned NKR-P1B^B6^ cDNA reported above (see also below) (37, 39). *Nkrp1e* contains an early stop codon in exon-3, and splicing of intron-5 is predicted to create a frame-shift in the open reading frame (ORF), suggesting it may represent a pseudogene, at least in the B6-strain (38). The *Nkrp1f* gene appears to be intact and is predicted to code for a functional protein. Work in our lab has identified the latest mouse family member, *Nkrp1g* (21, 39) (Figure 1).

Subsequent investigation into the nature of strain-dependent NK1.1 reactivity showed that it was due, at least in part, to a single amino acid substitution in the NKR-P1B gene products (and presumably the NKR-P1C gene products, although this remains to be shown) (39). The more recent development of NKR-P1B^B6^ (NKR-P1D) mAb (18, 40) has shown that, unlike NKR-P1C, which is expressed uniformly by all NK cells in B6 mice, NKR-P1B is variegated and only expressed at high levels on a subset of mature NK cells (36, 37, 41). Previous Southern blot analyses and more recent aCGH analyses, paired with phenotypic NK subset comparisons, have shown this trend to be a generalized phenomenon across many strains (34, 42).

**Figure 2:**
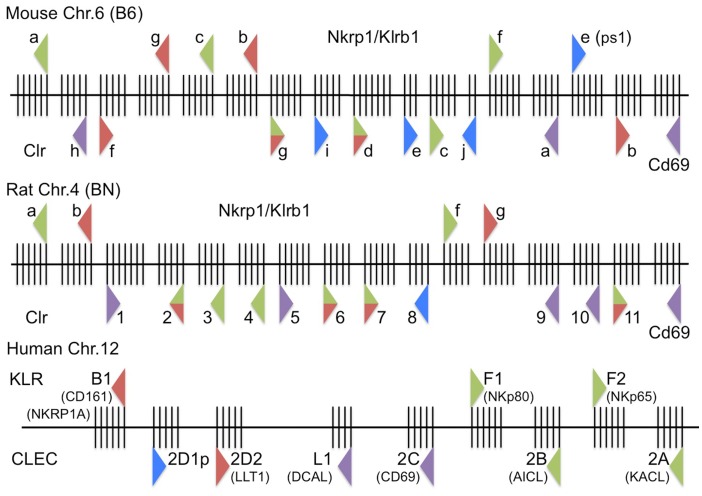
**Updated high-resolution maps of the rodent and human Nkrp1/*KLRB/F* and Clr/*CLEC2* receptor–ligand systems**. (Top) The mouse Nkrp1/*Klrb1* receptor genes and Clr/*Clec2* ligand genes on mouse chromosome 6 (B6-strain reference sequence, genome build: December 2011, GRCm38/mm10) are depicted, along with their orientation (triangle direction), known or suspected function (triangle color), and intron–exon structure (vertical exon lines); triangle representations are as in Figure 1. (Middle) The rat Nkrp1/*Klrb1* receptor genes and Clr/*Clec2* ligand genes on rat chromosome 4 (BN-strain reference sequence, genome build: November 2004, Baylor 3.4/rn4) are depicted as above. (Bottom) The human *KLRB/F* receptor genes and *CLEC* ligand genes on human chromosome 12 (genome build: February 2009, GRCh37/hg19) are depicted as above. Not to scale.

A recent comprehensive study by Hao et al. provided an in-depth computational analysis of the NKC regions from many species, including the rat *Nkrp1* genes (7). The rat NKC has at least four *Nkrp1* genes, predicted to represent orthologs of the *Nkrp1a/c, Nkrp1b/d, Nkrp1f*, and *Nkrp1g* gene sets (Figure 2). Like *Nkrp1b^B6^*, the rat *Nkrp1c* gene likely represents a divergent *Nkrp1b* allele present in the PVG rat strain (*Nkrp1b^PVG^*; see below) (43). While the activating function of the rat NKR-P1A receptor was known since the late 1980s, the inhibitory function of the rat NKR-P1B isoform was not shown until much later. Here, the Miller lab transfected human YTS cells with a rat cDNA library and sorted cells using the 10/78 mAb (similar NKR-P1A reactivity to 3.2.3 mAb) (44). Sequence analysis of positive clones fell into two groups corresponding to either rat NKR-P1A or NKR-P1B. Using chromium release assays, they showed that the rat NKR-P1B clones inhibited NK cytotoxicity. It is now clear that both the NKR-P1A/B gene products from some rat strains react with the 10/78 and 3.2.3 mAb, demonstrating that polymorphisms also exist in selected rat *Nkrp1* loci (45).

The closest human homolog, NKR-P1A (CD161/*KLRB1*), was identified in 1994 through the development of the DX1 mAb against human LAK cells (46). Human NKR-P1A shares 46% homology at the amino acid level with rat NKR-P1A and 46–47% homology with the mouse NKR-P1 proteins, but significantly lower homology with other C-type lectin-like NK cell receptors. Ligation of human NKR-P1A with DX1 mAb did not induce cytotoxicity against FcR-bearing P815 cells or have an effect on lysis of K562 target cells. However, when added to NK clones with the spontaneous ability to lyse P815 cells, cytotoxicity was inhibited, while F(ab′)_2_ of anti-NKR-P1A mAb did not inhibit, suggesting FcR cross-linking was important. Paradoxically, NK cell clones that did not spontaneously kill P815 targets could have a small proportion induced to kill P815 in the presence of NKR-P1A mAb. In 2005, two groups showed that the NKR-P1A receptor was indeed inhibitory on human NK cells, demonstrating it to be a likely functional homolog of the mouse and rat NKR-P1B receptors (47, 48). Also similar to the rodent NKR-P1B, only a subset of human NK cells express NKR-P1A (46). However, the existence of another putative inhibitory receptor in rodents, NKR-P1G, calls into question orthology of human NKR-P1A with rodent NKR-P1B (7). Whereas several different NKR-P1 transcripts could be detected by Northern blot in mouse NK cells (33, 49), only one band was detected in human NK cells (46). Nonetheless, an apparent lack of stimulatory NKR-P1 homologs in the human genome was recently re-investigated by the Steinle group, who suggested that the human *KLRF1/2* gene products, located telomeric to *CD69*, may actually represent divergent activating NKR-P1 homologs (see below) (23) (Figure 2).

### Discovery of the Clr (Clec2d/Ocil/LLT1) ligands

While investigating bone morphogenesis, Zhou et al. discovered a gene product they designated osteoclast inhibitory lectin (Ocil), after its ability to inhibit osteoclast formation when expressed on osteoblast cells (50). They went on to show that Ocil was a prototypical member of a group of similar gene products in mice (Ocil, Ocilrp1, Ocilrp2) (51). The extracellular domains of all three gene products are similar to each other at the amino acid (>70%) and nucleotide (~90%) levels. Around the same time, Plougastel et al. identified a novel set of *Clr* genes by sequencing B6-strain BACs of the NKC region centromeric to *Cd69* (52). The Clr gene products were shown to possess 40% amino acid identity to the lectin-like domain of CD69, and 80–90% homology at the nucleotide level to each other. They named the genes *Clr-a, -b, -c, -d, -e, -f, -g*, where *Clr-b* was identical to *Ocil, Clr-g* was identical to a splice variant of *Ocilrp2*, and *Clr-d* (and perhaps *Clr-c*) were similar to splice variants of *Ocilrp1* (51, 52). Since then, new family members, namely *Clr-h, Clr-i*, and *Clr-j*, were discovered by hybridizing partial *Clr* PCR products to mouse BALB/c and 129-strain BACs (21, 39) (Figure 1). While the majority of the *Clr* genes are predicted to code for functional proteins, *Clr-e* likely represents a pseudogene, due to a frame-shift (52, 53); *Clr-i* is a predicted pseudogene, due to multiple stop codons in all reading frames (21, 53); and *Clr-j* is only a gene fragment (53). Unfortunately, several nomenclature issues complicate the *Clr* literature, including synonyms and the existence of splice isoforms (see below) (Figure 1).

Subsequent to the identification of the *Ocil/Clr* gene family, several screens aimed at identifying NKR-P1 ligands demonstrated interactions between gene products of the two families [reviewed in Ref. (17)]. Briefly, interactions between the NKR-P1B/D:Clr-b as well as NKR-P1F:Clr-g receptor–ligand pairs were shown using cellular assays (involving BWZ.36 reporter cells bearing CD3ζ/NKR-P1 fusion receptors), NKR-P1 tetramers, and blocking mAbs for NKR-P1B^B6^ (a.k.a., NKR-P1D; 2D12 mAb) or Clr-b (4A6 mAb) (18, 20). These results were significant in that they showed an interaction between the NKR-P1 receptors, which were previously thought to recognize oligosaccharides (54, 55), and protein ligands. Thus, the NKR-P1 receptors are notable in that they recognize other Clr proteins encoded by the *Ocil/Clr/Clec2* ligand genes linked to the *Nkrp1/Klrb1* receptor genes. More recently, additional interactions have been shown in the mouse system, including strain-dependent conservation of NKR-P1B/D:Clr-b, NKR-P1F:Clr-c, -d, -g, and NKR-P1G:Clr-d, -f, -g (21) (Figure 1).

The first ligand identified in the rat system was a viral immunoevasin, RCTL, a spliced ORF with C-type lectin-like sequence homology identified in 2001 (56), derived from the rat cytomegalovirus-English isolate [RCMV-E; Mhv8 (57)]. *rctl* is expressed as an early gene upon infection of rat embryonic fibroblast (REF) cells (45, 56). The RCTL gene product appears to functionally replace the endogenous Clr-b-like ligand (rat Clec2d11), which is rapidly lost during RCMV-E infection; notably, both the host and viral ligands are recognized by specific allele(s) of the NKR-P1B receptor in certain rat strains (45). Prior to this work, no information was available regarding the rat Ocil/Clr gene family, until a breakthrough publication in 2006 by Hao et al. described the complete repertoire of NKC-associated *Clec*-like genes in several species (7). This in-depth analysis outlined at least 11 rat *Clec2d*-like genes or pseudogenes, making the rat system the most complex family identified to date. Due to close sequence homology and an inability to determine strict orthology with the mouse family members, the rat *Clr* genes were simply designated *Clec2d1-11* (centromeric to telomeric), with a close relative of mouse *Clr-b* (mouse *Clec2d8*) predicted to be rat *Clec2d11*, based upon sequence, structure, and genomic position (7). Indeed, rat Clec2d11 was shown to be recognized by two distinct host NKR-P1B alleles (45). In 2009, the promoter region of a rat *Ocil* homolog was first described (58); however, the gene annotated rat *Ocil* is another *Clr* family member, rat *Clec2d5* (7).

The first in-depth analysis of rat NKR-P1 ligands, which adopted a rat Clr-1–11 nomenclature based upon the corresponding Clec2d1–11 assignments, used an NFAT-driven GFP-reporter cell assay (BWN3G, similar to the BWZ.36 *LacZ*-reporter cell assay), to show that rat NKR-P1A and NKR-P1B (cluster 1 receptors) recognize Clr-11, while NKR-P1F and NKR-P1G (cluster 2 receptors) recognize an overlapping set of ligands, namely Clr-2, -3, -4, -6, -7 and Clr-2, -6, -7, respectively (59) (Figure 2). Follow-up work by this group showed that the mouse and rat NKR-P1F/G receptors are highly xenoreactive, cross-recognizing corresponding ligands from the other species and allowing some degree of assignment of functional orthology (22).

In the human system, a Clr sequence was discovered in the NKC in 1999 by Boles et al. by searching the EST database with a consensus sequence of known human C-type lectin-like receptors (60). They screened an NK cell cDNA library and identified an 850-bp cDNA, designated lectin-like transcript-1 (LLT1), and later generated a mouse anti-LLT1 mAb (L9.7) by immunizing AKR/J2 mice with LLT1 fusion proteins (61). LLT1 is similar in structure and function to the mouse Clr gene products, and indeed has been given the synonymous gene designation, *CLEC2D*, as the mouse Clr-b locus (*Clec2d*). Actually, Hao et al. identified two homologs, *CLEC2D1p* (a predicted pseudogene) and *CLEC2D2* (which encodes LLT1) (7) (Figure 2). However, LLT1 also shares significant homology with other rodent Clr gene products, as well as other human gene products, such as KACL (*CLEC2A*), AICL (*CLEC2B*), and CD69 (*CLEC2C*) (17, 23, 60, 62, 63). As with mouse Ocil/Clr-b, human OCIL was also discovered separately from LLT1 in 2004 (64), but the cDNA encodes an identical protein to LLT1; human LLT1/OCIL is 42% identical to mouse Ocil/Clr-b at the protein level.

The above features of LLT1 made it an attractive candidate for interaction with the human NKR-P1A receptor (61). Indeed, in 2005, simultaneous publications from two groups formally demonstrated an interaction between the human NKR-P1A (*KLRB1*) receptor and the LLT1 (*CLEC2D*) ligand, using LLT1 multimers and liposomes, reciprocal NKR-P1A/LLT1 reporter cell assays, as well as functional cytotoxicity and IFN-γ production assays (47, 48). More recent studies have identified several splice variants of the human *CLEC2D* gene, only one of which (LLT1) appears to functionally bind the human NKR-P1A receptor (65) (Oscar Aguilar; see GenBank KF958454-9, KF971856-9). As mentioned above, the Steinle lab has also reported significant *CLEC2* group homology between KACL (*CLEC2A*, linked to its receptor, *KLRF2*), AICL (*CLEC2B*, linked to its receptor, *KLRF1*), CD69 (*CLEC2C*, unknown receptor; between *CLEC2D* and *CLEC2A/B*), and LLT1 (*CLEC2D*, linked to its receptor, *KLRB1*) (23) (Figure 2).

## Nomenclature Issues and Functional Homology

The NKR-P1:Clr receptor–ligand system has historically suffered from considerable nomenclature issues, across different species as well as among distinct strains within a given species. In the mouse system, the receptors have been variably known as NK1/NK1.1 (an alloantigen between mouse strains), Ly-55(a–c)/Ly-59 (lymphocyte antigens), NKR-P1 (A–G, the common names), Cd161 (after the human receptor designation), *Klrb1* (the official gene nomenclature, with members *a–g*), and Clec5b (according to the C-type lectin-like nomenclature). While the official gene nomenclature could have been designated killer-cell lectin-like receptor group-B, with numerical family numbers (i.e., *Klrb1-7*; following the similar *Klra1-n* designations for Ly49a–x), it has instead adopted a hybrid lettered gene nomenclature, *Klrb1a–g*, after their common names. Thus, NKR-P1A is encoded by *Klrb1a*, etc. However, this is complicated by the fact that NKR-P1B and NKR-P1D are likely highly divergent alleles of the same or a similarly evolved single genetic locus; thus, NKR-P1B is encoded by *Klrb1b*, which is also known as *Klrb1d* in the B6 mouse strain (i.e., NKR-P1B^B6^ is synonymous with NKR-P1D). Following this, the remaining family members are NKR-P1C (encoded by *Klrb1c*), NKR-P1F (*Klrb1f*), and NKR-P1G (*Klrb1g*), plus one predicted pseudogene, NKR-P1E (*Klrb1e* or *Klrb1-ps1*) (53). While NKR-P1C^B6^ (*Klrb1c^B6^*) is widely thought to represent “the” NK1.1 antigen, it is actually only crossreactive as “one” NK1.1 antigen in the B6 and related mouse strains, as NKR-P1B alleles from several strains (e.g., NKR-P1B^Sw/SJL^) also encode NK1.1 antigens, and the NK1.1 mAb was originally derived by immunization of (C3H × BALB)F_1_ mice with CE-strain splenocytes and bone marrow (BM) cells (not B6 cells) (26). In addition, the *Nkrp1g* gene has also been referred to as a second *Nkrp1e*-like sequence (*Nkrp1e_2_*) (17), and most recently just *Klrb1*, perhaps signifying the closest predicted human *KLRB1* ortholog or functional homolog.

Furthering this complexity, as with any gene family, the discovery of the *Ocil/Clr* gene products by multiple groups has complicated the ligand nomenclature, which has been officially designated the *Clec2* gene family. Thus, the known mouse Clr ligands include Clr-a (encoded by *Clec2e*), Clr-b (*Clec2d*), Clr-c (*Clec2f*), Clr-d (*Clec2g*), Clr-f (*Clec2h*), and Clr-g (*Clec2i*). There are also a number of predicted pseudogenes, including Clr-e (*Clec2-ps1*; *Clec2d5*), three recently discovered members, Clr-h (*Clec2j*), Clr-i (*Clec2k*/*Clec2-ps2*), Clr-j (*Clec2-ps3*) (53), and an unlinked, more distantly related family member recently annotated Bacl (*Clec2l*), although Bacl is not encoded in the NKC (66). However, as mentioned above, under alternative nomenclature, other names have been assigned historically to the gene products: Clr-a (Clec2e, Clec2d7), Clr-b (Clec2d, Ocil, Clec2d8), Clr-c (Clec2f, Clr-z, Clec2d6), Clr-d (Clec2g, Ocilrp1, Ddv10, Clr-x, Clec2d4), Clr-e (Clec2-ps1, Clec2d5), Clr-f (Clec2h, Clec2d2), Clr-g (Clec2i, Ocilrp2, Dcl1, LCL-1(a–d), Clec2d3), Clr-h (Clec2j; Clec2d1), Clr-i (Clec2k, Clec2-ps2), and Clr-j (Clec2-ps3) (7, 53). Note that there are no annotated *Clec2a-c* genes in the mouse genome database, but these have been assigned to the human KACL (*CLEC2A*), AICL (*CLEC2B*), and CD69 (*CLEC2C*) loci. Also, note that an alternative myeloid receptor named Clec2 exists, which is distinct from the *Clec2* gene family and is encoded by the *Clec1b* gene (67). To date, many of the Clr have unknown receptors and most have unknown physiological function. However, the known mouse receptor:ligand pairs to date include NKR-P1B/D:Clr-b, NKR-P1F:Clr-c, -d, -g, and NKR-P1G:Clr-d, -f, -g in the B6, 129, and BALB/c strains (18, 20–22, 40) (Figure 1).

Adding to this diversity, the rat NKC contains 4 *Nkrp1/Klrb1* and 11 *Clr/Clec2* genes (7), and has had its share of nomenclature issues. The rat NKR-P1 receptors are fairly straightforward, and have been classified into two distinct structural and functional clusters, NKR-P1A/B (cluster 1), and NKR-P1F/G (cluster 2) (59). However, as in the mouse system, a highly divergent allele of the NKR-P1B receptor in the PVG strain (NKR-P1B^PVG^) has been alternately referred to as NKR-P1C (43). In addition, early references to NKR-P1F-like sequences were also called *Klrb1c*/NKR-P1C (68), and NKR-P1G-like sequences were also called *Klrb1d*/NKR-P1E. Notwithstanding this, the receptor designations have largely been resolved in recent literature. As above, human *KLRB1* may be more closely related to rat *Klrb1g* than *Klrb1b* (7), but this remains to be demonstrated.

Nonetheless, there are significant deviations in the ligand nomenclature, and due to the complexity and homology of the rat *Clec2* family, gene annotations are still being worked out in the genome browsers^1^, with many being given *RGD* or *LOC* designations (e.g., *RGD1564464/LOC500331* for *Clec2d1/Clr1*; see below). To nucleate the gene family, Hao et al. presented the earliest complete characterization of the rat *Clr* genes, and designated them numerically, centromere to telomere, as *Clec2d1–11* (7). There have been few updated attempts to rename these genes according to the mouse nomenclature, or to fully deduce speculated orthologies [if indeed possible (7, 69)], so they have simply been referred to as Clr-1–11 (22, 43, 59). However, the annotated rat *Ocil* gene is *Clec2d5* (Clr-5), distinct from a likely functional homolog of mouse Ocil/Clr-b, rat Clr-11 (*Clec2d11*), which is also the demonstrated rat NKR-P1B ligand (22, 45, 59). To confuse matters further, there is an annotated rat Clec2d-like-1 sequence (abbreviated Clec2dl1; not *Clec2d11*, or Clr-11) that is actually rat *Clec2d6*, or Clr-6. Notwithstanding these discrepancies, the known interactions in the rat system include: NKR-P1A/B:Clr-11, NKR-P1F:Clr-2, -3, -4, -6, -7, and NKR-P1G:Clr-2, -6, -7 in various rat strains (22, 45, 59). Interestingly, the mouse and rat NKR-P1F/G proteins have been labeled “promiscuous” because they share overlapping ligand specificity, cross-react in a xenogeneic manner with their respective ligands, and react with many rat tumor target cells (22). Indeed, the mouse NKR-P1F/G receptors can respond equivalently or better to their rat Clr ligand counterparts using functional reporter assays (e.g., mouse NKR-P1G also weakly binds rat Clr-4) (22) (Figure 2).

In humans, the system is relatively simple, apart from some synonymous designations and recent developments. The lone human NKR-P1A receptor (*KLRB1/CD161/CLEC5B*) possesses inhibitory function upon binding to the closest Clr-related ligand, LLT1 (*CLEC2D/OCIL/CLAX*) (47, 48). This interaction is functionally similar to the rodent NKR-P1B:Clr-b interaction; however, the receptor and ligand also share strong similarity with another rodent (putative) inhibitory interaction, NKR-P1G:Clr-d, -f, -g. Hao et al. originally identified two human *Clec2d* homologs, where *CLEC2D1p* is a predicted pseudogene (a.k.a., *LOC374443*), and *CLEC2D2* encodes LLT1 (7). However, recent work has speculated that the missing human functional equivalents of the rodent stimulatory NKR-P1 receptors may actually be located telomeric to CD69 (*CLEC2C*) as part of a greater *KLRB/F* family intermingled amongst *CLEC2* family ligands (7). Here, Nkp80 (*KLRF1*; *CLEC5C*) and NKp65 (*KLRF2*) are genetically linked with their respective ligands, AICL (*CLEC2B*) (62, 70) and KACL (*CLEC2A*) (63, 71). Of note, CD69 (*CLEC2C*) itself and a newly characterized non-NKC-encoded BACL (*CLEC2L*) have no known binding partners, but notably the latter is genetically linked with another lectin-like receptor, KLRG2 (*CLEC15B*) (66). There is also another more distantly related gene encoding DCAL (*CLECL1*), located between LLT1 (*CLEC2D*) and CD69 (*CLEC2C*) (Figure 2).

## Expression

### Mouse

Transcripts for all mouse *Nkrp1* genes have been detected in the spleen, thymus, lymph nodes, and other hematopoietic tissues, essentially wherever NK cells are found (53). Additionally, the lung and intestine have shown expression of almost all *Nkrp1* transcripts (53). *Nkrp1b/d* is unique in showing expression within the tongue and bladder (53). Within these tissues, *Nkrp1a, Nkrp1b/d, Nkrp1c*, and *Nkrp1f* transcripts (*Nkrp1g* has not yet been examined in detail) are present in NK cells, while *Nkrp1c* is also found in NKT cells (53). Subsets of cells classified by various sort markers as tissue DCs and macrophages have also been shown to express *Nkrp1b/d* (53). *Nkrp1f* transcripts are also present in BM DC/monocyte precursors and lymph node endothelial cells (53), and expression has also been reported in DC/APC (72, 73). *Nkrp1b/d* transcripts are also highly expressed in innate lymphoid cells (ILC), including ILC1, ILC2, ILC3, and LTi-like cells (David Allan, manuscript submitted). They are also enriched in a CD8αα^+^ subset of IEL; notably, these latter IEL also express greatly enriched levels of transcripts for *Nkrp1a*, upregulated almost 30-fold in CD8αα^+^ versus CD8αβ^+^ IEL (74). While *Nkrp1c* is found in almost all NK and NKT cells, expression of *Nkrp1b/d* is found at high levels in only a subset of NK cells (50–70%), but few NKT cells (~10%) (40) (see also Peter Chen et al., Munir Rahim et al., manuscripts submitted).

Consistent with the transcript data, most NKR-P1 receptors are expressed on mouse NK cells; however, few NKT cells express surface NKR-P1 receptors, with the exception of NKR-P1C, which is expressed at intermediate levels by NKT cells (40). Mouse NKR-P1B/D is only detected at significant levels on a subset of NK cells (~50–70%, depending on the strain), whereas the remainder expresses only low levels (18, 40). Strain-dependent NK1.1 expression is detectable on NK cells from a number of strains (CE, B6, BTBR, KK, NZB, NZW, C57L, C58, Ma/My, ST, SJL, FVB, and NIH-Swiss mice, but not 129, BALB/c, A/J, AKR, CBA, C3H, DBA, LG, PL, or SM mice); however, expression of the particular NK1.1 antigen involved (NKR-P1B or NKR-P1C) seems to be dictated by whether the majority of NK cells are NK1.1^+^ (NKR-P1C), or whether the NK1.1 expression is variegated (NKR-P1B), as determined by gating on DX5^+^ or NKp46^+^ NK cells (39, 40, 42). Surface expression of mouse NKR-P1G has not been documented yet, but it is predicted to be variegated, like NKR-P1B/D. Interestingly, both activating and inhibitory NKR-P1 isoforms are rapidly downregulated from the cell surface following ligation by plate-bound antibody or cognate ligands (40). On the other hand, surface NK1.1 (NKR-P1C) expression is induced on conventional CD8^+^ T cells following viral infection and activation by other stimuli (75–78). Expression of NK1.1 isoforms (NKR-P1B > NKR-P1C) is also found on immature thymocytes, thymic NK cells, fetal blood progenitors, subsets of mucosal T cells, and some NKp46^+^ gut ILC (36, 79–86).

Expression of the mouse *Clr* ligand genes is variable and highly regulated. Mouse *Ocil* (*Clr-b*) was reported to be expressed broadly in most tissues (52, 53), but is also prevalent during bone development and upregulated in response to osteotropic factors like retinoic acid, 1,25-dihydroxyvitamin D_3_, parathyroid hormone, IL-1α, and IL-11 (50). This regulation of *Ocil* appears to be unique, as *Ocilrp1* (*Clr-d*) and *Ocilrp2* (*Clr-g*) were not regulated by these treatments, despite *Ocilrp2* being co-localized in the same tissues as *Ocil* (51). At the transcript level, *Clr-a* is only expressed in the intestines, and even then only at low levels (53). *Clr-b* is expressed in all nucleated hematopoietic cells, some non-hematopoietic cells, and within most tissues, with the exception of the brain, which appears to be devoid of *Clr* and *Nkrp1* transcript expression (20, 50, 53). In addition, *Clr-b* transcript expression is tightly regulated on healthy versus pathological target cells. While expressed highly in normal cells, the transcripts are lost following transformation (20), viral infection (45), and genotoxic and cellular stress (87). *Clr-c* is expressed at low levels in the tongue, spleen, thymus, ovaries, testes, and lymph node tissues; *Clr-d* transcripts are uniquely present in the eye; and *Clr-f* is expressed in the liver and very highly in the kidney and intestine, specifically in intestinal epithelial cells and kidney tubular epithelial cells (52, 53). *Clr-g* is expressed in the spleen, thymus, and lymph node, particularly in intestinal epithelial cells, as well as LAK cells (52, 53); notably, distinct *Clr-g* splice isoforms exist (a.k.a., LCL-1a, -1b, -1c, -1d) that may have distinct expression patterns (72, 73). Expression of the remaining family members requires further investigation.

In terms of cell surface and protein expression, Clr-b has been the most widely studied, as detected using 4A6 mAb (20, 87). It appears to be a constitutive surface marker of healthy cells, as nearly all nucleated hematopoietic cells express it, as do normal embryonic and adult skin fibroblasts (20, 87). In line with this, Clr-b is frequently downregulated on pathological target cells, including most hematopoietic tumor cell lines (20), virally infected cells (e.g., MCMV, RCMV) (45), and cells undergoing genotoxic or cellular stress (87). Thus, like MHC-I molecules, Clr-b demonstrates a “missing-self” recognition pattern, expressed by healthy cells but lost on target cells undergoing pathological changes, making them more susceptible to NK cell effector function. Interestingly, the downregulation of Clr-b occurs at both the transcript and protein levels, suggesting that transcriptional, as well as post-transcriptional and post-translational mechanisms, play important roles in regulating surface Clr-b expression, and therefore regulating NK cell activity (45, 87). Notably, loss of cell surface Clr-b expression on stressed cells is abrogated by inhibitors of the ubiquitin–proteasome and endolysosomal pathways (e.g., MG132, lactacystin, chloroquine), many of which also affect autophagy (87). Expression of the other mouse Clr family members at the cell surface has been less documented due to a paucity of specific mAb.

### Rat

Where studied, expression in the rat system appears to be largely similar to that of the mouse. Rat NKR-P1A transcripts are found in NK cells and NKT cells, and within tissues that contain these cells (32). As in the mouse, the mAb that define rat NKR-P1 expression (3.2.3, 10/78 mAb) identify all NK cells from most strains, where they react with rat NKR-P1A, but these mAb also crossreact with the NKR-P1B inhibitory receptor on certain strains (44, 88). In PVG rats, two major subsets of NK cells can be phenotypically and functionally classified based upon their variegated expression of *Nkrp1b*: NKR-P1B^+^ NK cells (largely CD94/NKG2A^+^ but Ly49^−^); and Ly49s3^+^ NK cells (mostly NKR-P1B^−^ Ly49^+^ and responsive to MHC-I^−^ targets) (43, 89). This pattern is suggestive of subsets differentially regulated by MHC-I-dependent and MHC-I-independent recognition mechanisms. In addition, further characterization has revealed *Nkrp1b* to be highly expressed by unique subset(s) of circulating and liver/gut-resident lymphocytes, some of which may include mature/activated NK cells and/or ILC-like cells; these cells are enriched for CD8α, CD25, CD93, CX_3_CR1, but depleted for CD62L, CD27, and CD11b (90). Interestingly, *Nkrp1g* transcripts are exclusively expressed in the Ly49s3^+^ subset, while *Nkrp1f* is expressed in both subsets (22). This may be unique to the rat system, since mouse *Nkrp1*(*a, -c, -f, -g*) appear to be more equally expressed by the variegated *Nkrp1b*^+/−^ NK cell subsets (22). Little is known regarding the surface expression of the rat NKR-P1F and NKR-P1G receptors, although they are presumably expressed on the surface of NK cells. Expression on ILC subsets remains to be determined.

To date, little is known about the expression of the rat Clr molecules, in part due to a paucity of specific mAb. As seen with mouse Clr-b, rat Clr-11 (Clec2d11) is expressed broadly on *ex vivo* hematopoietic cells (as assessed using R3A8 mAb) (45). Moreover, expression is rapidly lost in REF cells following infection with RCMV-E (45). Whether or not this applies to other Clr family members needs to be tested; notably, expression of another Clr family member, Clec2d5, remained relatively unaffected or increased. General expression profiles of some of the rat Clrs have been inferred using qRT-PCR (69) or NKR-P1-bearing reporter cells (BWN3G) mixed with various types of stimulator cells (59). Ligand(s) for NKR-P1A/B^F344^ (most likely Clr-11) are expressed by *ex vivo* splenocytes, peritoneal exudate cells, and to a lesser degree on thymocytes, lymph node cells, and BM cells (59). This overlaps the expression visualized using reporter cells bearing the NKR-P1B^PVG^ and NKR-P1B^WAG^ alleles, which appear to respond more strongly and uniformly to most or all of the above cell types (especially BM cells) (45, 59). Similarly, NKR-P1F ligand(s) (most likely some combination of Clr-2, -3, -4, -6, -7) are expressed on peritoneal exudate cells, splenocytes, BM cells, thymocytes, and lymph node cells (59). NKR-P1G ligands (likely Clr-2, -6, -7) seem to be more restricted to peritoneal exudate cells, splenocytes, and BM cells (59). However, it should be kept in mind that an apparently restricted pattern may be partly due to non-uniform expression or a differential reactivity of rodent reporter cells expressing low or high-affinity receptors (e.g., NKR-P1B^F344^ versus NKR-P1B^PVG/WAG^) (45, 59). Interestingly, the RNK-16 rat NK cell line appears to express ligands for all the rat NKR-P1 receptors, while some tumor cells also express certain Clr ligands (22, 59). In one promoter study, binding sites for Sp1 family transcription factors such as Sp1 and Sp7 were shown to have an important role in the regulation of rat *Ocil* expression; however, the annotated rat *Ocil* gene is *Clec2d5* (Clr-5) not rat *Clec2d11* (Clr-11) (58).

### Human

Human NKR-P1A is expressed on the majority of (but not all) CD56^bright^ CD16^−^ and CD56^dim^ CD16^+^ NK cells, as well as subsets of CD4^+^ and CD8^+^ TCRαβ^+^ T cells, NKT cells, and TCRγδ^+^ T cells (48, 91, 92). It appears to be expressed on a higher proportion of memory versus naïve T cells (46, 93). Induction of NKR-P1A occurs in the fetal liver early in developmental ontogeny (46, 91), and it is one of the earliest markers of human NK cell development (94–97). It is also expressed *de novo* on CD34^−^ and CD34^+^ immature thymocytes, and is induced on thymocytes upon culture in rIL-2 (98). Surface NKR-P1A is upregulated on immature and mature NK cells upon exposure to IL-12 (94, 99, 100). NKR-P1A has also been reported to be a marker of all Th17 cell subsets [where it is induced by RORC (101)], human ILC and LTi subsets (102, 103), as well as a novel subset of FoxP3^+^ “Treg” found in both healthy and arthritic humans that secrete pro-inflammatory cytokines (104). CD4^+^ NKR-P1A^+^ T cells are capable of migration in transwell assays and transendothelial migration *in vitro* (105). Acquisition of NKR-P1A has also been reported to be an early event in monocyte differentiation and on DCs (106).

In contrast to mouse Clr-b and rat Clr-11, and perhaps more akin to other rodent Clr, human LLT1 transcripts are only expressed at low levels in NK cells, T cells, B cells, and osteoblasts, but are undetectable in monocytes (65, 91, 107). In addition, LLT1 is expressed in some tumor lines, and its expression is greatly increased by mitogen stimulation or activation of lymphocytes, including LAK cells, NK cells, T cells, B cells, and DC (65, 91, 107). Induction in osteoblasts has also been documented using IL-1α and PGE_2_ (64). While freshly isolated B cells and DCs express little LLT1 mRNA, transcript levels are augmented following infection with viral pathogens (e.g., influenza, HSV-1, EBV, HIV) or TLR agonists (i.e., TLR-3, -4, -7, -8, -9), in particular CpG DNA treatment (91, 107). Again unlike rodent Clr-b/Clr-11, LLT1 protein is not detected at the surface under naïve, resting conditions; however, mirroring transcript expression, surface LLT1 is highly upregulated upon activation with the stimuli noted above. Induction on B cells also occurs following IgM cross-linking or CD40 ligation. LLT1 expression is also inducible on T lymphocytes upon stimulation via CD3 cross-linking, PMA, PHA, and IL-2 (61, 91, 107). On DC, the most potent inducers of LLT1 expression are CpG DNA, polyI:C, LPS, TLR-7/8 agonists, and IFN-γ (107). LLT1 is also induced on NK cells upon activation, following incubation with certain NK-sensitive target cells (91, 107). It remains to be determined whether NKR-P1A:LLT1 interactions regulate NK and T cell functions *in cis*, in addition to recognition of target cells *in trans* (108, 109).

Expression of the other human CLEC2 ligands is more restricted. As the names suggest, KACL (*CLEC2A*) is expressed in keratinocytes, AICL (*CLEC2B*) is induced upon activation of hematopoietic cells, CD69 (a.k.a., EA-1/AIM; *CLEC2C*) is induced early after activation of T and NK lymphocytes and other hematopoietic cells, BACL (*CLEC2L*) is expressed in brain tissue, and the more distantly related DCAL (*CLECL1*) is expressed in dendritic cells (23, 110).

## Structure

### Genomic

In the mouse B6-strain reference genome, all of the *Klrb1* genes have six coding exons: exon-1 codes for the cytoplasmic domain, exon-2 for the transmembrane domain, exon-3 for the stalk region, and exons-4, -5, -6 for the extracellular lectin-like domain (21, 38). This gene structure appears to be conserved for the *Klrb1* genes in the BALB/c and 129 strains (53), human *KLRB1* (46), as well as the *Klrb1* genes in the rat BN-strain reference genome, with the exception of rat *Nkrp1f* (*Klrb1c/f*), which may have five to six exons, depending upon reference genome build (7) (Table 1). Notably, the human *KLRF1* and *KLRF2* genes also have a six-exon gene structure (Figure 2; Table 1).

**Table 1:** **Structure of the rodent and human Nkrp1-related genes (*Nkrp1/Klrb1*; *KLRB/F*)**.

Species	Gene	Exons	Size (kb)	Gene ID (Entrez) (Ref)
Mouse^a^	*Nkrp1a* (*Klrb1a*); *MGI107540*	6	13.7	17057
	*Nkrp1b* (*Klrb1b/d*); *MGI107539*	6	12.6	80782
	*Nkrp1c* (*Klrb1c*); *MGI107538*	6	10.2	17059
	*Nkrp1e* (*Klrb1-ps1*); *MGI3624540*	6	12.9	724020
	*Nkrp1f* (*Klrb1f*); *MGI2442965*	6	11.5	232408
	*Nkrp1g* (*Klrb1/1g*); *MGI96877*	6	16.5	100043861
Rat^a^	*Nkrp1a* (*Klrb1a*); *RGD1586149*	6	16.7	362443
	*Nkrp1b* (*Klrb1b*); *RGD1583688*	6	12.4	678513
	*Nkrp1f* (*Klrb1c/f*); *RGD1587571/1583336*^b^	5–6^b^	4.1–11.6^b^	689809; 683758^b^
	*Nkrp1g* (*Klrb1/1d/1g*); *RGD1587563*	6	18.7	689817
Human^a^	*KLRB1* (*NKR-P1A*/CD161); *HGNC6373*	6	12.6	3820
	*KLRF1* (NKp80); *HGNC13342*	6	17.5	51348
	*KLRF2* (NKp65); *HGNC37646*	6	14.3	100431172

*^a^N.B., based upon the following genome builds: mouse B6-strain reference sequence, December 2011, GRCm38/mm10; rat BN-strain reference sequence, November 2004, Baylor 3.4/rn4; human genome reference sequence, February 2009, GRCh37/hg19*.

*^b^Ambiguity exists: rat genome build, March 2012, RGSC 5.0/rn5*.

The *Clec2* genes in the mouse and rat also have a similar genomic structure. All the genes have five exons, with the following exceptions: mouse *Clrg* (*Clec2i*, six exons); mouse *Clrd* (*Clec2g*, seven exons); mouse *Clre* (*Clec2-ps1*, three exons); mouse *Clrj* (*Clec2-ps3*, two exons); rat *Clr1* (*Clec2d1*, seven exons); rat *Clr5* (*Clec2d5*, five to six exons); rat *Clr7* (*Clec2d7*, six exons); rat *Clr8* (*Clec2d8p*, four exons); and rat *Clr9* (*Clec2d9*, six exons) (Table 2). The human *CLEC2D* gene that encodes LLT1 (a.k.a., *CLEC2D2*) also has five exons, as does the upstream *CLEC2D1p* pseudogene (7). The human *CLEC2A* and *CLEC2B* genes also possess a five-exon gene structure, and the same is true for the *CLEC2C* gene that encodes CD69 (23, 110). The recently described *CLEC2L* (*BACL*) gene, which is not linked to the NKC, also possesses five exons, but the less related *CLECL1* (*DCAL*) gene linked to the NKC only has four exons (Figure 2; Table 2).

**Table 2:** **Structure of the rodent and human C-type lectin-related genes (*Clr/Clec2*; *CLEC2*)**.

Species	Gene	Exons	Size (kb)	Gene ID (Entrez) (Ref)
Mouse	*Clra* (*Clec2e*); *MGI3028921*	5	9.0	232409
	*Clrb* (*Clec2d*); *MGI2135589*	5	5.9	93694
	*Clrc* (*Clec2f*); *MGI3522133*	5	6.4	435921
	*Clrd* (*Clec2g*); *MGI1918059*	7	50.3	70809
	*Clre* (*Clec2-ps1*)	3	13	(53)
	*Clrf* (*Clec2h*); *MGI2136934*	5	14.9	94071
	*Clrg* (*Clec2i*); *MGI2136650*	6	9.2	93675
	*Clrh* (*Clec2j*); *MGI3647940*	5–6^b^	4.6–9^b^	677440 (53)
	*Clri* (*Clec2-ps2*)	5	29	(53)
	*Clrj* (*Clec2-ps3*)	2	1	(53)
Rat	*Clr1* (*Clec2d1*); *RGD1564464*	7	14.7	500331
	*Clr2* (*Clec2d2*); *RGD1562831*	5	8.1	362445
	*Clr3* (*Clec2d3*); *RGD1588731*	5	9.1	689757
	*Clr4* (*Clec2d4*); *RGD1588718*	5	17.4	689770
	*Clr5* (*Clec2d5*^a^); *RGD620070*	5–6^b^	10.0	113937
	*Clr6* (*Clec2d6^a^*); *RGD1588698*	5	7.3	689790
	*Clr7* (*Clec2d7*); *RGD1587580*	6	33.7	689800
	*Clr8* (*Clec2d8p*)	4	9	(7)
	*Clr9* (*Clec2d9^a^*); *RGD1306865*	6	106.1	312745
	*Clr10* (*Clec2d10^a^*); *RGD1587527*	5	5.3	689853
	*Clr11* (*Clec2d11^a^*); *RGD1563148*	5	8.6	362447
Human	*CLEC2A* (KACL); *HGNC24191*	5	19.1	387836
	*CLEC2B* (AICL); *HGNC2053*	5	17.5	9976
	*CLEC2C* (CD69); *HGNC1694*	5	8.4	969
	*CLEC2D1p* (*LOC374443*)	5	41.1	374443
	*CLEC2D2* (LLT1); *HGNC14351*	5	29.8	29121
	*CLEC2L* (BACL); *HGNC21969*	5	21.1	154790
	*CLECL1* (DCAL); *HGNC24462*	5	17.4	160365

*^a^Alternate names: rat *Clec2d5* (*Ocil*; *Clec2d*); rat *Clec2d6* (*Clec2dl1*); rat *Clec2d9* (*Clec2h*); rat *Clec2d10* (*Clec2e*); rat *Clec2d11* (*Clec2g*). Based upon genome builds outlined in Table 1*.

*^b^Ambiguities exist*.

The promoter regions of the rodent *Nkrp1* and *Clec2* genes remain poorly characterized. There were early attempts to analyze the upstream regions of the mouse *Nkrp1* genes in different strains (49, 111). The proximal promoters appear to be TATA-less, relying on initiator (Inr) and downstream promoter elements (DPE) (111). The mouse *Nkrp1* genes also appear to possess a three-promoter organization similar to that seen for the mouse *Ly49* genes (112). Several predicted transcription factor-binding sites in the distal and proximal promoter regions are speculated to be important in regulating *Nkrp1* expression, including consensus Ets-1, Ikaros, GATA, TCF-1, Sp1, NFAT, and Oct-1 sites (111). In addition, a number of upstream regulatory elements have been mapped, including transcriptional start sites (TSS), alternative exons, and a distal DNase hypersensitive site that may function as an enhancer element. Luciferase-based reporter assays have demonstrated that a 600-bp core promoter drives expression in both T/NK lineage cells, while a 9.76-kb upstream region enhances expression in NK-like cells but represses expression in pre-T cells, suggesting lineage-specific regulation (111). Northern blot analysis has revealed differential expression of *Nkrp1a-c* transcripts in select strains, but culture of LAK cells in IL-2 could induce *Nkrp1* expression where low (49).

In the mouse Clr system, *Ocil/Clrb* and *Ocilrp2/Clrg* have been reported to possess an inverted TATA box upstream of their TSS, while *Ocilrp1/Clrd* has a more traditional TATA box (51). Both *Ocilrp1/Clrd* and *Ocilrp2/Clrg* have been reported to generate alternative splice variants (51), and an independent characterization of *LCL-1* (*Ocilrp2/Clrg*) transcripts suggests that up to four different splice variants exist (*a–d*) (72, 73). The human *OCIL* (*CLEC2D/LLT1*) gene lacks an inverted TATA sequence, instead containing a GAATCA sequence upstream of the TSS (64). The human *CLEC2D* gene has also been reported to generate several alternative splice variants (other than LLT1), some of which may be functional (e.g., some code for proteins retained in the ER as heterodimers with LLT1, while others lack a transmembrane domain) (65) (Oscar Aguilar, see GenBank KF958454-9, KF971856-9). As mentioned above, Sp1 transcription factor-binding sites (Sp1, Sp7) were shown to regulate expression of the rat *Ocil* gene (*Clec2d5*; *Clr-5*) (58).

### Protein

The NKR-P1 and Clr proteins belong to group-V of the 14 C-type lectin superfamily groups (7, 113). C-type lectins are a class of glycoprotein characterized by their Ca^2+^ dependence, conserved disulfide-linked cysteine residues, and functional carbohydrate recognition domains (113). The NKR-P1 and Clr proteins are designated as “lectin-like” because they primarily bind other proteins, and they possess somewhat atypical conservation of their cysteine residues, such that they may have lost the residues and loop structures required to coordinate divalent calcium ions, and thus may lack high-affinity Ca^2+^-dependent binding to carbohydrates. For example, the mouse Clr and human LLT1 are all missing the C5 residue in their lectin-like domain, and the C4 residue is also absent from mouse Clr-b and Clr-g (52). The implications of these changes require further investigation. However, it should be noted that the NKR-P1 receptors were first reported to possess functional carbohydrate recognition domains (54, 55). In addition, the human LLT1/OCIL and mouse Clr-b/Ocil proteins have been reported to bind high-molecular weight carbohydrates (including sulfated forms) in a Ca^2+^-independent manner, blockable using soluble carbohydrates (including heparin and chondroitin sulfate) (64).

Initial work on the structure of the mouse NKR-P1 proteins demonstrated that they likely exist as disulfide-linked homodimers. For example, NKR-P1D (NKR-P1B^B6^) is ~90 kDa under non-reducing conditions, but ~47 kDa under reducing conditions, when assessed using 2D12 mAb (18). The Clr proteins also likely exist as homodimers, but heterodimers between different Clr have also been speculated to exist (18). Crystal structures for the mouse NKR-P1 proteins are just beginning to be published and assessed. Although early co-crystal structures were first reported in 2003 for the mouse NKR-P1F:Clr-g and NKR-P1D:Clr-b proteins (the latter reported under conditions requiring mutagenesis of certain residues), these have not been published (18). Published work suggests that mouse NKR-P1A^B6^ and NKR-P1C^B6^ have a similar structure, with slight differences explainable by their amino acid sequences (e.g., NKR-P1A^B6^ has a higher Y residue content than NKR-P1C^B6^) (114). Mouse NKR-P1C^B6^ also has a better-defined conformation of disulfide bridges than NKR-P1A^B6^, suggesting that NKR-P1C is more rigid and stable; indeed, this is supported in the literature regarding their individual stable expression at the cell surface on NK cells (40). However, it should be noted that the NKR-P1C^BALB^ receptor lacks a potentially crucial conserved C4 residue (position C122S) that may have implications for its expression and function, in addition to its lack of NK1.1 reactivity due to a S191T substitution (which is also found in the functional NKR-P1B^BALB^ receptor) (39, 115).

Rat NKR-P1A and NKR-P1B protein structures have also been compared. Interestingly, rat NKR-P1A is more similar to mouse NKR-P1A/C than to rat NKR-P1B (114). The rat NKR-P1B protein has more α-helical content (including an additional α-helix) than rat NKR-P1A. The human NKR-P1A protein has been modeled on these rodent NKR-P1 structures, where it more closely resembles NKR-P1B than other isoforms, in that it is predicted to retain an additional α-helix (114).

In general, all of the NKR-P1 proteins seem to possess a fold similar to other C-type lectin-like domains, including at least two α-helices and two anti-parallel β-sheets. They have a β-core composed of long anti-parallel β-strands that form a central pillar, flanked at one end by short β-strands and at the other end by a β-sheet (114). This core is surrounded by α-helices (two to three, depending on the isoform). All appear to have three disulfide bonds, except mouse NKR-P1C^BALB^, which has only two (114). Comparison of all known NKR-P1 ectodomains revealed the highest conservation in the β-core, less conservation in the loop regions, with the longest loop suggested to play a role in ligand specificity (114). A mouse NKR-P1A crystal structure reported recently suggests that a claw-like structure may be important for ligand interaction (116).

A recent biochemical study of the human NKR-P1A:LLT1 proteins have shown that they interact with a *K*_d_ ~ 50 μM (117). Reciprocal mutagenesis of the proteins also provided a partially validated structural model of the interaction, supporting a dimeric interaction with several important contact residues. Even more recent co-crystal structures of the high-affinity (*K*_d_ ~ 2 nM) human NKp65:KACL interaction have suggested a conserved docking topology for the interaction of genetically linked C-type lectin-like receptor–ligand pairs in the NKC (118). Here, two NKp65 receptor monomers, with limited dimeric interface between them, contact a single dimeric KACL ligand bivalently at two distinct but symmetrical sites in a butterfly-shaped complex. A similarity in this study was noted to the compact Ly49C dimer interacting independently with two monomeric H-2K^b^ MHC-I ligands (119). Whether or not this applies to other NKC-encoded receptor–ligand pairs remains to be elucidated, but the remaining genetically linked receptor–ligand interactions have all been shown to interact with much lower affinities (micromolar versus nanomolar) (118).

Much less is known about the structure of the rodent Clr or human LLT1 proteins. Human LLT1 has been modeled on a CD69 structural backbone (65), and models have also been proposed based upon mutagenesis studies (117), as well as the human NKp65:KACL crystal structure (118). Recently, the structure and biophysical properties of mouse Clr-g were determined by expressing soluble portions of the extracellular lectin-like domain (120). In this structure, Clr-g exists as a monomer or dimer, depending on the size of the ectodomain used. In terms of secondary structure, it has 2 α-helices, 2 anti-parallel β-sheets composed of 3 β-strands each, and 2 disulfide bonds, with a dimeric interface involving 10 hydrogen bonds. Mouse Clr-g has mostly positive electrostatic potential, while its cognate receptor, NKR-P1F, has negative potential, suggesting electrostatic potential may be a driving force in their contact (120). Notably, models of the other Clr, and other C-type lectin-like proteins, are predicted to possess distinct surface electrostatic potential in this work. Mouse Clr-g also shares almost ~47% sequence identity with human KACL, and predictive models of most of the human and mouse NKR-P1:Clr receptor–ligand interactions have been outlined based upon the human NKp65:KACL interaction (118).

## Function

### Signaling

Signaling via NK cell receptors is hypothesized to deliver either an activating signal or to exert inhibitory effects on downstream signaling cascades, and the NKR-P1 family is no exception to this notion of paired or balanced recognition systems. Early experiments demonstrated that Syk acts as a common signaling element important for both FcR-initiated and natural cytotoxicity in NK cells (121). However, additional pathways involving Src-family and PI3K kinases are also involved in triggering natural cytotoxicity (122–124).

In this light, it was shown that the mouse NKR-P1C receptor activates both redirected cytotoxicity and IFN-γ production in NK and NKT cells, and that association with the FcRγ adaptor protein was crucial for these functions (28, 125). Furthermore, while FcRγ homodimers were shown to be required for both optimal NKR-P1C expression and signaling in both NK cells and NKT cells (125), FcRγ/CD3ζ heterodimers may be sufficient for residual NKR-P1C cell surface expression, in contrast to CD16 expression, which is dependent upon FcRγ homodimers and is negatively regulated by CD3ζ (via FcRγ/CD3ζ heterodimers) (126). In addition, the rat NKR-P1A receptor was shown to induce Ca^2+^ flux, phosphatidylinositol turnover, PLA_2_ activation, arachidonic acid release, heterotrimeric G-protein activation, redirected cytotoxicity, and granule exocytosis by rat NK cells and the RNK-16 cell line (127–129). Subsequent analysis of a number of NKR-P1 protein sequences revealed the presence of potential PLC-γ1/2 motifs (YxxL), SH3-binding proline-rich domains, Lck-recruitment motifs (CxCPR/H), and variably present consensus ITIM motifs (ΦxYxxΦ) in the rodent but not human NKR-P1 receptors (68). The activating rodent NKR-P1 receptors were later shown to contain a conserved positively charged amino acid residue (R) in their transmembrane domain, likely for FcRγ adaptor association (based upon a distinct transmembrane R amino acid position versus K for DAP12-associated receptors), while the inhibitory rodent NKR-P1 receptors contain consensus ITIM motifs (36, 68, 130).

Notably, the rodent NKR-P1 CxCPR/H motifs, similar to those found in the CD4 and CD8 T cell co-receptors, were shown to functionally recruit Lck by immunoprecipitation, yeast two-hybrid analysis, and functional mutagenesis (131, 132). In addition, Lck was also shown to be necessary for efficient NKR-P1C-mediated redirected lysis, as cytotoxicity was diminished in BM-derived LAK cells from Lck^−/−^ mice (whereas splenic LAK cells may use another Src-family member to compensate) (132). The ITIM motif in the mouse NKR-P1B receptor was also shown to recruit the SHP-1 phosphatase upon pervanadate stimulation (and to a lesser extent SHP-2) (36, 132). A consensus ITIM motif (133) is also present in the mouse and rat NKR-P1G receptors, which are presumably inhibitory; however, mouse NKR-P1G lacks the consensus Lck-recruitment motif (21). The functional consequences of the loss of the di-cysteine motif remain unknown, but may suggest a more direct inhibitory receptor function versus a co-inhibitory receptor function, if the CD4/8 T cell co-receptors provide any reference. The mouse and rat NKR-P1F receptors may either be stimulatory, co-stimulatory, or function as co-receptors in terms of signaling (40), as they retain a charged transmembrane residue, yet mouse NKR-P1F lacks the conserved YxxL motif, instead containing a Y residue in a different position and context (21).

Like the rat NKR-P1A receptor (127), the mouse NKR-P1C receptor (NK1.1 in B6 mice) has also been shown to signal IFN-γ production in part via PI3K (p110γ > δ) activity (134), suggestive of some co-stimulatory function, akin to NKG2D isoforms (135–138). Given the specific expression of NKR-P1C on NKT cells [but not other mouse NKR-P1 receptors (40)], its ability to recruit Lck (akin to a T cell co-receptor), and its partial signaling via the PI3K pathway (similar to CD28, NKG2D), it could serve as a co-receptor or costimulatory receptor (signal 2) for TCR activation (signal 1) on NKT cells in their recognition of CD1d-restricted ligands. Since the mouse NKR-P1C ligand(s) remain unknown, it is possible that endogenous or foreign glycolipids (and/or glycoproteins) could be recognized simultaneously by the NKT cell TCR and this lectin-like receptor to facilitate dual recognition (co-receptor or accessory function) and/or co-stimulation. Unlike conventional MHC-restricted CD4^+^ and CD8^+^ T cells, NKT cells are not yet known to express a co-receptor for CD1, although they are tightly regulated by SAP/SLAM family interactions (13, 14). On the other hand, conventional NK cells express most NKR-P1 receptor isoforms [whereas NKT cells more uniquely express NKR-P1C (40)], and CD1d over-expression on target cells has been reported to inhibit NK cell function via an as yet unidentified receptor (139, 140).

Interestingly, the cytoplasmic tail of the human NKR-P1A receptor contains neither a strong consensus ITIM motif (ΦxYxxΦ) (133), nor an intact Lck-binding motif (CxCPR/H) (68). Nonetheless, the human NKR-P1A cytoplasmic tail does contain a tyrosine residue in an atypical motif (AxYxxL) that may function as a weak ITIM (48, 68), and the receptor has been shown by immunoprecipitation and Western blotting to form large complexes containing the Src-family kinases, Lck, Fyn, and Lyn in Brij-58-solubilized NK cells (141). However, association with SHP-1 has not been demonstrated (47, 48), and Lck association was not observed in CD161^+^ NKT cells (142). The receptor has been shown by yeast two-hybrid and co-immunoprecipitation studies to associate with acid sphingomyelinase (aSM) in primary human cell lines and NK cells (143). Ligation using CD161 mAb results in association of CD161 with aSM in detergent-resistant membranes, activation of aSM activity, production of ceramide, PI3K-dependent activation of the Akt and ERK pathways, and proliferation (143). Ligation of human NKR-P1A on thymocytes has been reported to inhibit cytotoxicity but enhance proliferation (98), expression on T cells enhances transendothelial migration (105), and ligation on macrophages and DC fluxes intracellular calcium and leads to IL-1α and IL-12 production (106). More recently, human NKR-P1A has also been shown to be expressed specifically by Th17 cells (101, 144), MR1-restricted MAIT cells (145), and a unique “Treg” subset (104), where its function(s) are still being evaluated (92).

### Autoimmunity and disease association

Human NKR-P1A was shown to be preferentially expressed on γδ T cells expressing the Vδ2 chain (93). Interestingly, the proportion of γδ T cells and IL-17^+^ T cells expressing NKR-P1A is higher in MS patients than in healthy donors (93, 146). Upregulation of human NKR-P1A on γδ T cells also occurs following exposure to IL-12, as it does for NK cells, but only for the Vδ2^+^ population, because Vδ1^+^ cells express lower levels of the IL-12Rβ_2_ subunit (93). These NKR-P1A^+^Vδ2^+^ T cells also undergo transendothelial migration in an NKR-P1A-dependent manner *in vitro*. Since MS patients have more of these T cells, it has been proposed that they use NKR-P1A to migrate to local lymph nodes, recirculate, and extravasate into the brain, leading to exacerbated MS symptoms (93).

NKR-P1A^+^ T cells can also be detected in inflammatory infiltrates in patients suffering from psoriasis (144) and Crohn’s disease (147), with 20-fold more NKR-P1A^+^CD4^+^ T cells present in patients with Crohn’s disease than healthy patients (147). Whether this is due to upregulation of NKR-P1A on reactive T cells, or exacerbated production or expansion of pre-existing NKR-P1A^+^ Th17 cells, requires further investigation (92). Importantly, however, an inflammatory bowel disease (IBD) susceptibility SNP in the human LLT1/OCIL (*CLEC2D*) gene was recently found to be associated with Crohn’s disease, but not ulcerative colitis (148).

Interestingly, an N19K substitution in the human LLT1/OCIL (*CLEC2D*) gene has been found to be associated with reduced bone mineral density in postmenopausal women (149). Genome-wide association studies should reveal new interactions between this receptor–ligand system and human disease.

### Viral infection

Viral evasion of host innate and adaptive immunity is a well-documented evolutionary occurrence. In 2001, a spliced C-type lectin-like ORF was identified in the RCMV-E genome (56). The gene product was designated RCTL, and it was later shown to closely resemble the coding sequence of an endogenous rat *Clec2d*-like sequence, *Clec2d11*, a predicted functional homolog of mouse Clr-b (*Clec2d*) (45). Indeed, an mAb specific for the RCTL protein (R3A8 mAb) was also shown to crossreact with the host rat Clr-11 (Clec2d11) protein. Expression of *rctl* was detected as early as 3 h post-infection (characterizing it as an early gene), while expression of host rat *Clec2d11* was reciprocally lost from the cell surface, replaced by RCTL (although heterodimers of host Clec2d11 and RCTL may exist). BWZ.36 reporter cells expressing a CD3ζ/RCTL fusion receptor responded strongly to 293T transfectants expressing the rat NKR-P1B receptor (indeed, only the NKR-P1B^WAG^ allele), suggesting a direct interaction between RCTL and the inhibitory NK cell receptor. RCTL expression on infected cells moderately inhibited WAG-strain LAK cell activity, an effect that was abrogated with either R3A8 mAb blockade or infection using an RCMV ΔRCTL-mutant virus (45). The ΔRCTL-mutant virus exhibited lower splenic and liver titers *in vivo*, but only in rat strains where the RCTL protein interacted strongly with the NKR-P1B allele. Notably, weak recognition of RCTL was also shown by the activating rat NKR-P1A receptor, suggesting that the host and pathogen were evolving and counter-evolving this innate recognition mechanism. Recently, downregulation of Clr-b has also been shown to occur upon infection with poxviruses, including vaccinia virus and ectromelia virus, where loss of the surface protein was dependent upon active virus infection but not late viral gene synthesis (150). It will be interesting to investigate the roles of similar CMV-encoded genes with lectin-like homology [e.g., *r153* in RCMV-Maastricht (151)] in NK cell recognition, as well as whether these CMV immunoevasins are capable of heterodimerizing with host proteins (e.g., rat Clec2d1–11).

Induction of LLT1 surface expression has been shown to occur upon infection with several viruses (e.g., EBV, HIV, influenza, HSV) (91, 107). In addition, LLT1 induction by TLR agonists (e.g., TLR3, 4, 7, 8, 9) as well as other immune stimuli (such as TCR/BCR cross-linking, mitogens, CD40, etc.) suggests that LLT1 interaction with human NKR-P1A also regulates immune responses during infection, particularly viral and bacterial infections (91, 107). In this light, several lectin-like viral ORF (including those from vaccinia and other poxviruses) have been speculated to play roles in the regulation of host immune responses, but none of these have been evaluated to date (56).

### T cell co-stimulation and adaptive immunity

Few reports to date have examined the effects of the rodent NKR-P1:Clr receptor–ligand system on the adaptive immune response. Ligation of an Ocilrp2 splice isoform (LCL-1; Clr-g) expressed on activated T cells by NKR-P1F on B cells and DC has been reported in mice, leading to enhanced TCR/CD28-mediated T cell proliferation and IL-2 production (73). However, the topology of this interaction would not be predicted by the somewhat restricted expression of NKR-P1F mainly within NK and T cells, with splice variants of Clr-g being more broadly expressed (53). Thus, in light of the recently identified “promiscuous” NKR-P1F:Clr-c, -d, -g and NKR-P1G:Clr-d, -f, -g interactions (21), the reagents used in these studies may possess multiple specificities (e.g., polyclonal anti-Clr-g Ab could cross-react with other Clr, Ocilrp2–Ig fusion protein could recognize both NKR-P1F/G, NKR-P1F tetramer could recognize Clr-c/d/g). Nonetheless, Clr-g was reported to be induced more rapidly after CD3 cross-linking than other co-stimulatory molecules (e.g., OX40, 4-1BB) (73). Also, the co-stimulatory effect of Clr-g was shown to require and synergize with CD28, while OX40 and 4-1BB can act independently of CD28 (73). In a follow-up study, silencing of Clr-g resulted in impaired T cell proliferation and IL-2 production, due to an inability of the responding T cells to phosphorylate Lck, reorganize the actin cytoskeleton, form TCR caps, and transduce NFκB signals (72). These studies highlight an important issue regarding the perceived roles of “receptor” versus “ligand” (or vice versa) in NKR-P1:Clr interactions (51). Notably, the mouse Clr-b protein has been suggested to possess a TRAF2-interaction motif (130), supporting the possibility of “reverse signaling” in regulation of NFκB signals by the Clr proteins on target cells and APC, but the function of this motif has not been investigated yet.

In the human system, CD161 on T cells may function as a costimulatory receptor or accessory molecule for recognition of CD1d (142). Ligation of CD161 on NKT cells did not directly activate cytokine secretion (IFN-γ, IL-4), nor cause proliferation. However, co-ligation of CD3 and CD161 could augment cytokine production and proliferation, and this could be diminished using soluble CD161 mAb. Similarly, while LLT1 inhibits cytotoxicity and IFN-γ production via NKR-P1A on NK cells, it appears to costimulate IFN-γ production by NKT cells (47, 107). Reciprocal studies have suggested that LLT1 itself may signal on NK and NKT cells to augment IFN-γ production via a Src-family–ERK signaling pathway (61, 152).

### Ribozyme

A recent publication examined the presence of discontinuous hammerhead ribozymes in mammalian genomes and identified several such self-cleaving RNA motifs within the 3′-UTRs of mouse *Clec2e* (Clr-a), mouse *Clec2d* (Clr-b), and rat *Clec2d11* (Clr-b-like), along with others in the 3′-UTRs of predicted *Clec2*-like genes in other species (153). Discontinuous hammerhead ribozymes differ from other ribozymes in that they exist as two fragments (enzyme, substrate) *in cis*, separated by an insertion of variable length sequence into the stem-1 loop, rather than being contiguous [reviewed in Ref. (154–156)]. In the mouse *Clec2d*, mouse *Clec2e*, and rat *Clec2d11* gene products, this insertion varies from ~250 to ~690 to ~790 nucleotides, respectively, and serves to separate the substrate sequence (upstream) from the enzyme sequence (downstream) (153). In addition, these ribozyme sequences were shown to be catalytically active (with the mouse *Clec2d* ribozyme being more active than the *Clec2e* ribozyme), reducing luciferase reporter expression by ~80% due to autocatalytic reporter RNA degradation (153). The net effect of these cleavage events would be predicted to separate the mRNA 5′-cap and coding sequence from the poly-A tail of the transcript, leading to altered RNA stability. This suggests another possible level of regulation (post-transcriptional) of the rodent *Clec2d* and *Clec2e* gene products, either enhancing mRNA turnover to provide more efficient “missing-self” recognition, or providing a molecular switch to turn on and off expression of the gene products during immune responses.

Interestingly, other closely related *Clec2* genes contain substrate-like motifs in their 3′-UTRs (in the absence of corresponding enzyme motifs), each with similar sequence identity to the substrates in *Clec2d* and *Clec2e* (153). It would be interesting to see whether these sequences can act in *trans* as substrates for either the *Clec2d* or *Clec2e* enzyme components, akin to micro-RNA-mediated regulation.

### Evolution

It is clear that the NKR-P1:Clr (*Klrb:Clec2*) systems, although genetically linked, have undergone significant evolution between species and between strains within a given species. It has been proposed that the creation and deletion of Ly49 receptor genes can occur by unequal crossing over during meiosis, generating new inhibitory or stimulatory Ly49 by swapping the cytosolic and transmembrane exons (which encode function) with the ectodomain exons (which dictate specificity), even incorporating pseudogene exons (17). The NKR-P1:Clr system is no exception to this form of evolution, and the head-to-tail orientation of some genes suggests how this may occur. Notably, the *Nkrp1b* and *Nkrp1c* genes are arranged such that even a continuous transcript may be possible, splicing together the inhibitory region of NKR-P1B and the NK1.1^+^ ectodomain of NKR-P1C, generating a novel inhibitory receptor that is NK1.1^+^ with new affinity and/or specificity in certain mouse strains (Figure 2). This is also possible for other *Nkrp1* receptor and *Clr* ligand genes in mice, rats, and perhaps humans. This arrangement would be ideal for counter-evolving stimulatory receptors to directly recognize viral decoys that inhibit NK cell function by co-opting self ligands, or for modulating the specificity of self-specific inhibitory receptors to counter–evade interaction with such decoys (45). In addition, the promiscuity of the NKR-P1F/G receptors for overlapping ligands may have evolved under selection pressure from viral evasion strategies. Whether or not additional novel viral immunoevasins exist that may target the NKR-P1:Clr systems is currently under investigation.

## Conflict of Interest Statement

The authors declare that the research was conducted in the absence of any commercial or financial relationships that could be construed as a potential conflict of interest.

## Footnotes

^1^http://genome.ucsc.edu; http://www.ncbi.nlm.nih.gov
